# Label-free single-vesicle based surface enhanced Raman spectroscopy: A robust approach for investigating the biomolecular composition of small extracellular vesicles

**DOI:** 10.1371/journal.pone.0305418

**Published:** 2024-06-18

**Authors:** Zirui Liu, Martin Ng, Siddharth Srivastava, Tieyi Li, Jun Liu, Tuan Anh Phu, Bogdan Mateescu, Yi-Ting Wang, Chia-Feng Tsai, Tao Liu, Robert L. Raffai, Ya-Hong Xie

**Affiliations:** 1 Department of Materials Science and Engineering, University of California Los Angeles, Los Angeles, California, United States of America; 2 Northern California Institute for Research and Education, San Francisco, California, United States of America; 3 Brain Research Institute, University of Zürich, Zürich, Switzerland; 4 Institute for Chemical and Bioengineering, ETH Zürich, Zürich, Switzerland; 5 Biological Sciences Division, Pacific Northwest National Laboratory, Richland, Washington, United States of America; 6 Division of Vascular and Endovascular Surgery, Department of Surgery, University of California San Francisco, San Francisco, California, United States of America; 7 Department of Veterans Affairs, Surgical Service (112G), San Francisco VA Medical Center, San Francisco, California, United States of America; 8 UCLA Jonsson Comprehensive Cancer Center, University of California, Los Angeles, Los Angeles, California, United States of America; National University of Singapore - Kent Ridge Campus: National University of Singapore, SINGAPORE

## Abstract

Small extracellular vesicles (sEVs) are cell-released vesicles ranging from 30-150nm in size. They have garnered increasing attention because of their potential for both the diagnosis and treatment of disease. The diversity of sEVs derives from their biological composition and cargo content. Currently, the isolation of sEV subpopulations is primarily based on bio-physical and affinity-based approaches. Since a standardized definition for sEV subpopulations is yet to be fully established, it is important to further investigate the correlation between the biomolecular composition of sEVs and their physical properties. In this study, we employed a platform combining single-vesicle surface-enhanced Raman spectroscopy (SERS) and machine learning to examine individual sEVs isolated by size-exclusion chromatography (SEC). The biomolecular composition of each vesicle examined was reflected by its corresponding SERS spectral features (biomolecular “fingerprints”), with their roots in the composition of their collective Raman-active bonds. Origins of the SERS spectral features were validated through a comparative analysis between SERS and mass spectrometry (MS). SERS fingerprinting of individual vesicles was effective in overcoming the challenges posed by EV population averaging, allowing for the possibility of analyzing the variations in biomolecular composition between the vesicles of similar and/or different sizes. Using this approach, we uncovered that each of the size-based fractions of sEVs contained particles with predominantly similar SERS spectral features. Indeed, more than 84% of the vesicles residing within a particular group were clearly distinguishable from that of the other EV sub-populations, despite some spectral variations within each sub-population. Our results suggest the possibility that size-based EV fractionation methods produce samples where similarly eluted sEVs are correlated with their respective biochemical contents, as reflected by their SERS spectra. Our findings therefore highlight the possibility that the biogenesis and respective biological functionalities of the various sEV fractions may be inherently different.

## Introduction

sEVs, commonly referred to as exosomes, are cell-released vesicles ranging in size from 30-150nm. This class of EVs are increasingly a focus of intense investigation in all biomedical fields because of their potential to serve as diagnostic tools and therapeutics for diseases [1–6]. Studies have revealed the heterogeneity among sEVs, leading to the interest in understanding the biological relevance of specific sEV subpopulations [7–9]. Studies investigating sEV subpopulations have been focusing on the variation in specific surface markers or cargo molecules of different vesicle groups isolated based on various physical properties [10–15]. In addition, understanding the heterogeneity of sEVs at the single-vesicle level provides a complementary perspective and has the potential to recover information lost when looking at population averages.

Currently, common methodologies used for the isolation and fractionation of sEVs are based on their biophysical properties, e.g., their size or density using techniques such as ultracentrifugation, density gradient ultracentrifugation (DGUC), and size exclusion chromatography (SEC). Each of these techniques have their advantages and limitations [16–18]. Ultracentrifugation remains one of the most commonly used methods for the isolation of sEVs [19, 20]. However, it is challenging for conventional ultracentrifugation to effectively isolate sEV subpopulations as all EVs are collectively isolated in a pellet, a process that has been shown to cause EV aggregations [21–23]. Recent studies have led to improvements in ultracentrifugation, especially by combining density gradient ultracentrifugation and iso-osmotic gradient ultracentrifugation, methods that have been shown to resolve subpopulations of EVs in a more gentle and reproducible manner [24–26].

With SEC, a solution (mobile phase) is run through a porous filtration matrix fixed within a column (stationary phase), resulting in distinct fractions [27]. Using this approach, larger particles elute before smaller ones, which gently fractionates sEVs using size as the criterion. In addition to the relatively rapid processing time and low cost, SEC possesses the advantage of preserving the structure and integrity of sEVs after their fractionation [28–32]. Therefore, SEC is widely used by laboratories to isolate sEV subpopulations for downstream biochemical analyses.

Currently, several approaches are commonly used for the biomolecular characterization of sEVs. Western blot is still regarded as a gold standard for protein detection and analyses in biological samples and it is still widely used in EV studies. However, Western blot is a bulk-based detection method. Typical experiments require more than 10^6^ sEVs for one analysis [33]. Therefore, distinctive features associated with individual types of sEVs are likely lost during the procedure. Though studies have been published on using Western blot for the biomolecular characterization of content within subpopulations of sEVs, limitations of the approach impede discovering the homogeneity or heterogeneity within each of the EV size groups due to the limit of detection of the technique [34].

In addition to Western blotting, enzyme linked immunosorbent assay (ELISA) is another popular characterization method for identifying unique markers on the surface of sEVs (e.g., CD63 & CD9). As an antibody-based technique, ELISA provides marker information with high specificity [35–37]. However, the process normally requires the determination of the target surface marker prior to the analysis, limiting the ability to explore biomolecular discrepancies among sEV subpopulations. Also, non-specific cross-reactivity often causes noise that can adversely impact the specificity and limit of detection of the approach [38].

Vesicle flow cytometry (vFC), which uses fluorescently-labelled antibodies, has been attracting interest because it has single vesicle sensitivity and the capability to provide biochemical information due to the specificity between antibody and sEV surface antigens [39, 40]. Studies with next-generation cytometers have attempted to improve the limit of detection of conventional systems, which ranges from around 100nm to 300nm, in order to improve the study of smaller vesicles like sEVs [41–43]. Moreover, flow cytometry is capable of running in a “label-free” format to determine the size and concentration of the EVs. Although vFC offers a powerful approach for detecting target surface markers on the vesicle samples, it shares limitations associated with Western blot and ELISA. This includes a need for knowing the targets prior to processing the sEV samples, which poses challenges to new marker discovery, which in turn hinders the study of the heterogeneity at the single-vesicle level [44, 45].

Genomic studies of EV cargo often make use of unbiased sequencing and polymerase chain reaction (PCR) to analyze their oligonucleotide content [46]. These approaches provide an alternative to surface marker-based characterization techniques with the capability of providing molecular content information for the vesicles [47, 48]. Similar to Western blot, PCR requires lysing of the sEV samples before initiating the process. This imposes the impossible challenge of retaining the unique biochemical information from individual sEVs intact for identification [49, 50].

While technologies reviewed here offer opportunities for detecting and analyzing sEVs for specific applications, they share challenges that limit their ability to fully identify and understand sEV subpopulations. Such hurdles must be overcome in order to address the question of how a population of sEVs reveal their biomolecular composition discrepancies from a single vesicle perspective. Among alternative technologies detailed above, we propose Raman spectroscopy/SERS, as it has the ability to address some of the unmet needs of current biomolecular-based sEV assessment approaches.

Raman spectroscopy offers the ability to identify biochemical substances through their structural fingerprints. It does so by interrogating the unique chemical bonding strengths within molecules that are reflected by a laser beam interacting with the substances, commonly referred to as Raman scattering [51, 52]. Raman spectroscopy has an inherent ability to reveal differences in the chemical composition of different specimens. However, the chance for Raman scattering to happen unaided is extremely low (roughly 10^−6^) [53]. In order to boost the Raman spectral signal of a substance, metallic nanostructures are often utilized to trigger surface-enhanced Raman spectroscopy (SERS) [54]. The nanoscopic areas on the metallic nanostructures where the Raman signal is highly enhanced are called “hotspots”. The SERS enhancement will quickly dissipate from the hotspot with distance [55].

Recently, a number of publications have revealed the potential of SERS in analyzing EVs, including sEVs. Penders et al. (2021) developed a Raman trapping technique of single EVs to establish the breast cancer cell-derived EVs as biomarkers [56]. Kruglik et al. (2019) advanced a Raman tweezer capable of analyzing sEVs at a diameter of 100nm [57]. Shin et al. (2020) combined SERS of sEVs and deep learning for early stage lung cancer detection with area under the curve (AUC) > 0.9 [58]. Dong et al. (2020) reported that SERS spectral variation of protein phosphorylation inside the vesicles could serve as an indicator for detecting four types of cancers [59]. To prevent the drying of vesicle solutions during the measurements, Rojalin et al. (2020) developed a porous scaffold SERS platform, resulting a clear spectral distinguishability between cancer patients and healthy individuals [60]. In addition to detecting the spectral features directly, Banaei et al. (2021) modified SERS substrate for vesicle immobilization, which allows for quantitative analysis via variations of the SERS indicator intensities [61].

We previously reported the design of a unique SERS platform based on gold nanopyramids, which allowed for efficient biological substance detection at a single-molecule level of sensitivity [62, 63]. Interestingly, the “hotspot” size of our platform is comparable to the size of individual sEVs (~80-100nm) and located at the ladders of each of the nanopyramids [62, 63]. With the quadratic dependence on the intensity of the local electromagnetic field, SERS signal from one single hotspot dominates. Together with the hotspot size, it suggests that on average a SERS spectrum embodies information derived from the entire volume of individual vesicles when applied to our platform detecting sEVs [64, 65]. We successfully applied this platform to examine sEVs from different sources, demonstrating proof-of-concept for the use of our method to detect single sEVs in a sensitive manner that could distinguish between different sEV sources [65]. Our previous publications have illustrated the capability of SERS detection of single sEVs as a non-destructive spectroscopy method that can provide comprehensive biochemical information of sEVs. It has the potential to reveal differences among individual sEVs, making our method suitable for sEV subpopulation determination.

In this study, we combined the use of our SERS gold nanopyramid substrate along with the use of a customized machine learning program to deconvolute the spectral signatures collected from individual sEVs fractionated by SEC. To this end, we made use of a well-defined HEK293 cell line and its HRAS transgenic variant cell line as parental cells to derive vesicles of interest. The HRAS transgene, when expressed in one of the HEK293 cell lines, led to EVs that were enriched with a membrane-bound fluorescent mNeonGreen protein. For our study, which used HRAS-positive and HRAS-negative HEK293 cells, EVs were isolated from the conditioned cell culture media using cushioned-ultracentrifugation, which served to gently concentrate the sEVs, and subsequently fractionated using SEC [24]. To establish the rigor of this novel approach, we compared the SERS data with mass spectrometry (MS) data obtained from the same samples of sEVs.

Results of our study reveal that the SERS spectral signatures correlate with the biomolecular composition of sEVs. The findings are supported by SERS spectra that were analyzed using linear discriminant analysis (LDA). SERS spectral signatures collected from vesicles in different SEC fractions revealed marked differences with small overlaps, suggesting that some of the sEVs in different fractions shared a common biomolecular composition. This was achieved despite the non-zero spectral feature variations within each of the subpopulations, likely caused by an inherent biological variability among individual EVs. Collectively, results of our study lay the foundation for future investigations into the relationship between sEVs derived from SEC and their biomolecular composition at the single EV level.

## Materials and methods

*sEV isolation and nanoparticle tracking analysis (NTA)* sEVs were isolated from conditioned cell culture medium by first performing Cushioned-Ultracentrifugation (C-UC) [24], followed by SEC using IZON 35 nm qEV columns. More detailed descriptions of the approaches can be found in *Supporting Information*.

*Immunoblotting* The detailed descriptions of the immunoblotting used in this study can be found in *Supporting Information*.

*Transmission electron microscopy* sEV morphology was assessed by loading 5 μL of a sample onto a glow-discharged 300 mesh Formvar-coated copper grid. The particles were left to settle for 2 minutes, and then excess moisture was wicked away. The grids were then washed 3 times with 1% uranyl acetate (UA), after which the grid was left to rest on a drop of 1% UA for 1 minute. Grids were imaged at 120kV using a Tecnai 12 Transmission Electron Microscope (FEI) at the EM-Lab at the University of California, Berkeley.

*SERS substrate fabrication* This study used SERS platforms fabricated according to the same method as we previously reported [62]. Detailed description of the fabrication can be found in *Supporting Information*.

*Raman spectroscopy* First, 5 μL of each sEV sample solution was dropped onto the SERS substrate using a micropipette and dried. Raman measurements were performed using the Reinshaw inVia Raman spectrometer at room temperature. The laser excitation wavelength was 785 nm. The power used was 5 mW. 500x optical microscope was used for focusing the laser beam, resulting in a ~1 μm laser spot diameter. Before measuring sEVs, the system was calibrated using the 520 cm^-1^ peak of silicon. The exposure time was 0.2 s to avoid sample overheating. To collectin SERS spectra from multiple sEVs, a Raman mapping measurement was performed over a 1.2 mm × 1.2 mm square with respect to the center of each sample droplet. One spectrum was collected from each of the data spots and the step width was 5 μm to avoid double collecting, thus over-fitting in spectral analysis. The detection range of Raman shifts was from 564 cm^-1^ to 1681 cm^-1^.

*Scanning electron microscopy (SEM)* SEM used in this study was Nova 230. The working distance was ~5.0mm. The acceleration voltage was 10 kilovolts. The images were taken between 45,000× and 55,000× magnification. The electron detector used was TLD (through the lens) detector to obtain the signal from the secondary electrons.

*MS-based proteomics analysis* The detailed protocol of the MS proteomics analysis used in this study can be found in *Supporting Information*.

*SERS spectral data analysis* For spectral analysis, algorithms were adopted from the open source libraries of Numpy, Pandas, Sklearn, and Scipy. To generate the simulated spectra based on MS, the relative abundance of proteins obtained by MS (90 in total) were first translated to the relative abundances of the 20 amino acids inside the human body, using web-scraping of the Uniprot online database. The signal-to-noise ratios were used as an indicator of SERS activity. Combining the signal-to-noise ratios and the MS derived relative abundance, 20 independent coefficients for the 20 amino acids were obtained, respectively. These coefficients were used to obtain a linear combination of the 20 SERS spectra, which is the final simulated SERS spectrum. To generate the fitted spectrum, Adaboost was used. The dataset included 20 average SERS spectrum of amino acids as data instances, and 1117 points of Raman shift as the features. The fitting coefficients from Adaboost were then compared with the normalized relative-abundance ratios obtained from MS, and we obtained a mean-deviation of less than 10%. Linear Discriminant Analysis (LDA) for the dimensionality reduction and supervised clustering, accommodating the 1117 dimensions corresponding to the 1117 data points on the Raman shift axis. The 1117 dimensions were reduced to 1 dimension in the case of a binary cluster system (for e.g., HEK293 vs HEK293+HRAS), and 2 dimensions for (for e.g., IZON SEC fractions 7, 8, 9). The LDA model was trained on the following dataset– 1117 Raman shifts as features, and each SERS spectrum as a data Instance. The trained model was used to transform the existing data set to a reduced dimension dataset for the purpose of visualization. The transformed data was plotted in 2 dimensions, and the results are presented in two dimensional plots.

## Results and discussion

**Fig 1:**
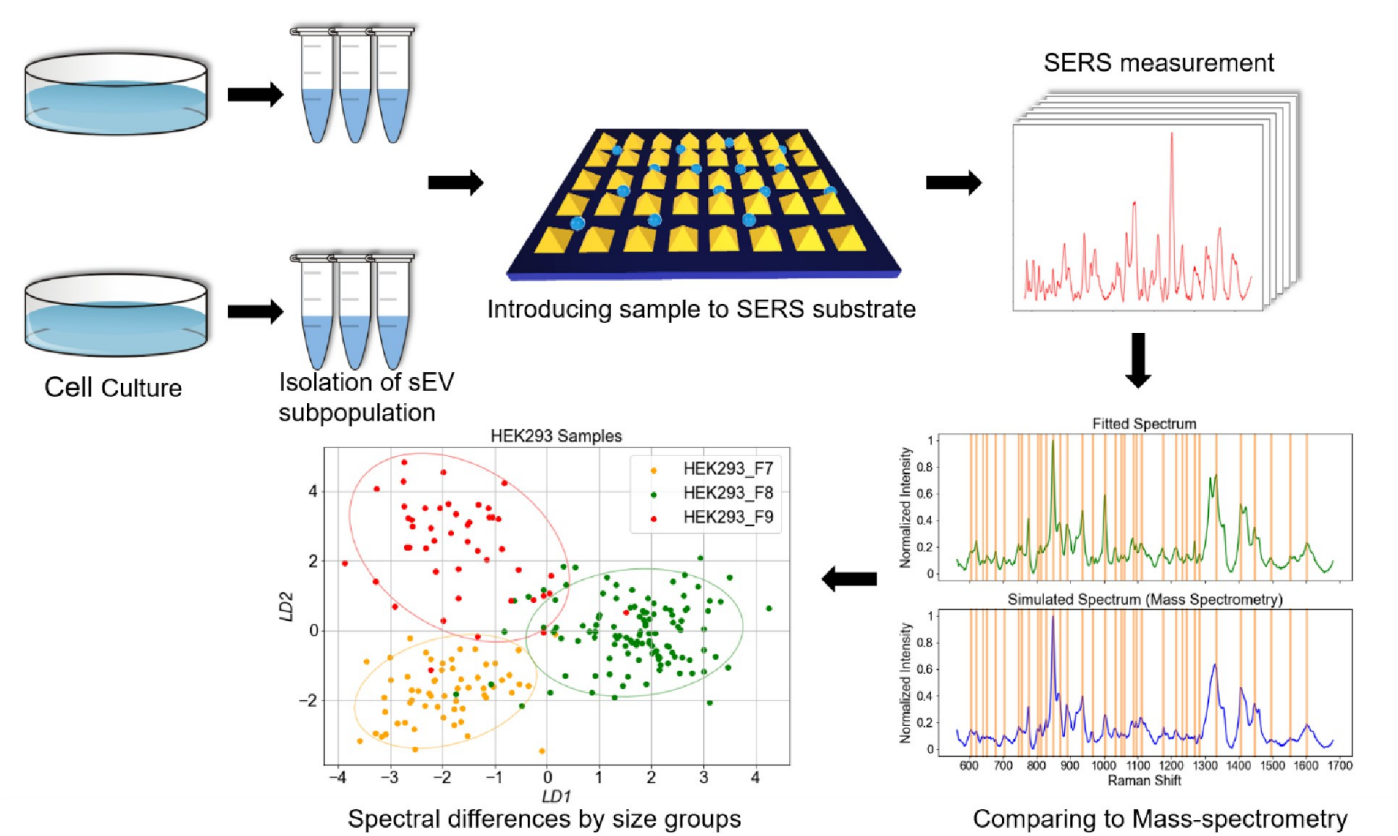
Schematic of the experimental flow in this study.

**Fig 2:**
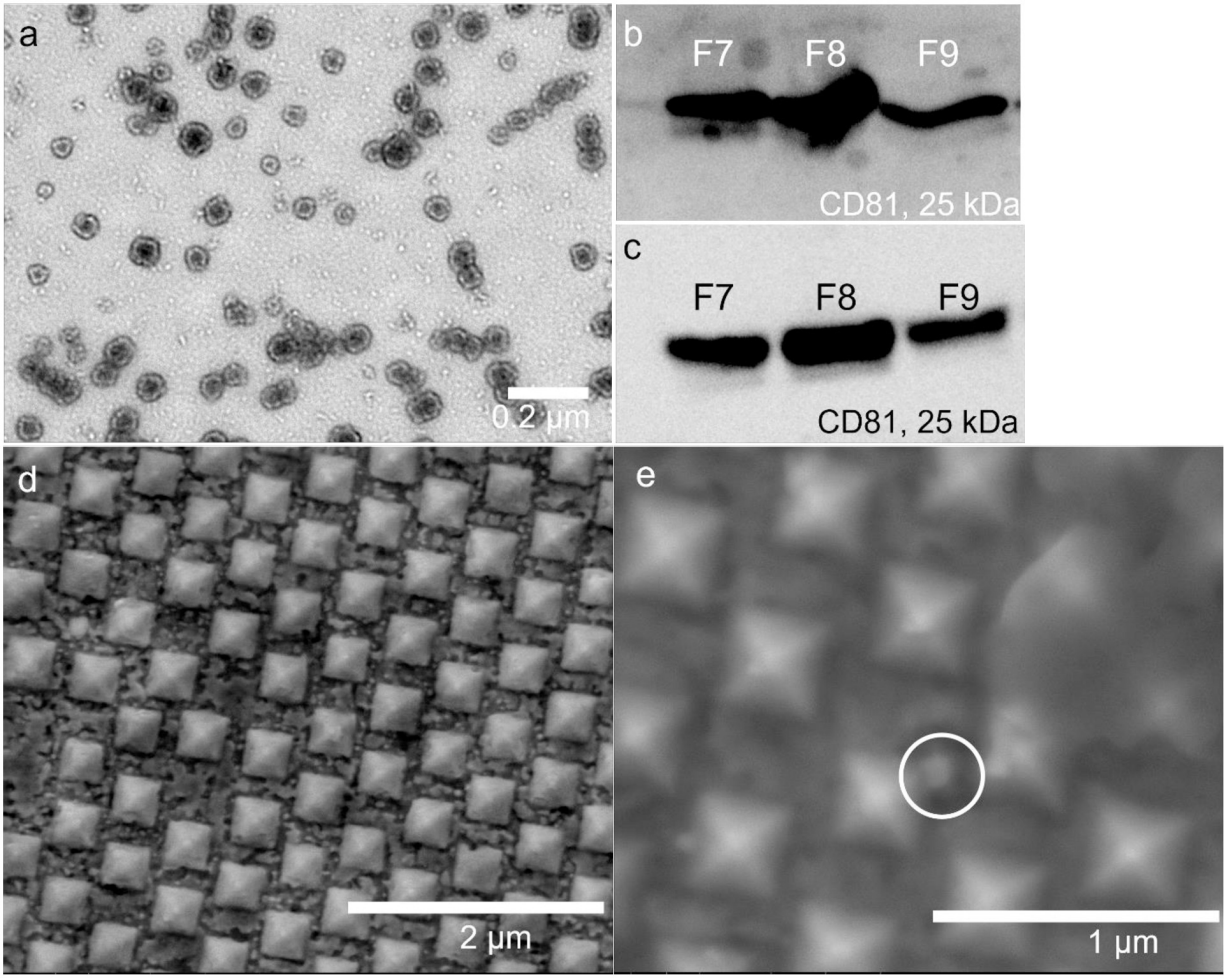
Overview of the vesicle samples and the SERS substrate. (a) TEM image of the sEVs; (b) Western blot of HEK293 derived vesicles; (c) Western blot of HEK293+HRAS derived vesicles; (d) SEM image of the SERS substrate; (e) SEM image of the SERS substrate after sample introduction.

The experimental flow of this study is shown in Fig 1. sEVs were first concentrated and isolated from conditioned cell culture mediums of HEK293 and HEK293+HRAS cells. Subsequently, sEVs were sub-fractionated using SEC with qEV IZON columns. Purified sEVs resided mainly within three fractions: 7, 8 and 9 (F7, F8, F9 in short). As shown in Fig 2b & 2c, Western blot analyses confirmed the presence of CD81 positive sEVs derived from the cultured HEK293 and HEK293+HRAS cells respectively. Our results also verified that the vesicles, fractionated by SEC from the respective conditioned medium of HEK293 and HEK293+HRAS cells, eluted in different fractions indicating size differences in the sEV subpopulations. The NTA measurements of sEVs derived from SEC fractions 7, 8, 9 in each of the cell types are shown in S1-S6 Figs in S1 File for vesicles prepared from both cell types. An EM image of the vesicles is shown in Fig 2a. The lipid bilayer of the particles can be clearly observed, and the average sizes fell within the commonly accepted range of sEVs. Fig 2, together with S1-S6 Figs in S1 File, present a comprehensive overview of the sEVs examined in this study. Detailed procedures can be found in supporting information.

We next focused on these sEV-rich subfractions (7, 8, 9) for their assessment using SERS. To this end, a small volume of each fraction (5 μL) was carefully dropped onto SERS gold nano-pyramid substrates before taking the SERS measurements. One SERS spectrum was collected from each vesicle within a defined sub-fraction. A comparison between the SERS data and MS data was performed to verify that the SERS spectra was contributed by the biomolecular composition of the sEVs. Subsequently, the SERS data was analyzed by LDA to explore a possible correlation between the size-based sEV fractions and their biomolecular composition-based discriminations.

Data in Fig 2d & 2e illustrate representative SEM images of the SERS substrates before (2d) and after (2e) the introduction of sEV samples. The nano-pyramids are clearly visible in images taken for both conditions. Findings shown in Fig 2e show the interaction between the substrate and the sEVs. Furthermore, dendrites visualized on top of the nano-pyramids were attributed to phosphate buffered saline (PBS) crystals that had formed during the drying process of the sample solution. On the top right portion of Fig 2e, an example of PBS crystals covering the nano-pyramids can be seen. The charging effect observed on the SERS substrate caused by PBS crystals prevented the acquisition of high-resolution SEM images of the structures.

Prior to analyzing individual sEVs isolated using SEC, we first focused on determining how well the SERS spectra collected from individual sEVs correlated with their biomolecular compositions. Although the use of Raman/SERS is recognized as a powerful tool in determining the compositional “fingerprint” of biological substances, its capacity to interrogate sEVs has not been well established yet. Therefore, based on the physical principles of Raman/SERS, we inferred that SERS spectra from the collective of Raman-active chemical bonds inside sEVs could serve to resolve the biomolecular composition of individual vesicles. We sought to test this idea by correlating the sEV SERS spectral data to their proteomic analysis by classical mass spectrometry (MS). The primary sEV samples used for this application were from cultured HEK293+HRAS fraction 8. We chose this fraction as it contained the most abundant amount of sEVs as detected by NTA. It should be pointed out that such a comparison between SERS and MS is qualitative rather than quantitative, meaning that we carried out such a comparison based on the existence of a biological species or the SERS peak locations from a spectral perspective. This is because SERS quantification is yet to be fully established [66]. At the same time, it is challenging for MS to provide absolute quantitative information during discovery of peptides/proteins without authentic standards [67].

Following the collection of MS data, which revealed the amino acid frequency in the sEVs (S1 Table in S1 File), we verified how this correlated to the natural abundance of the 20 amino acids within proteins that commonly occur in human tissues. Subsequently, we recorded the SERS spectra for all 20 amino acids (purchased from Sigma Aldrich) and present our findings in S7 Fig in S1 File.

**Fig 3:**
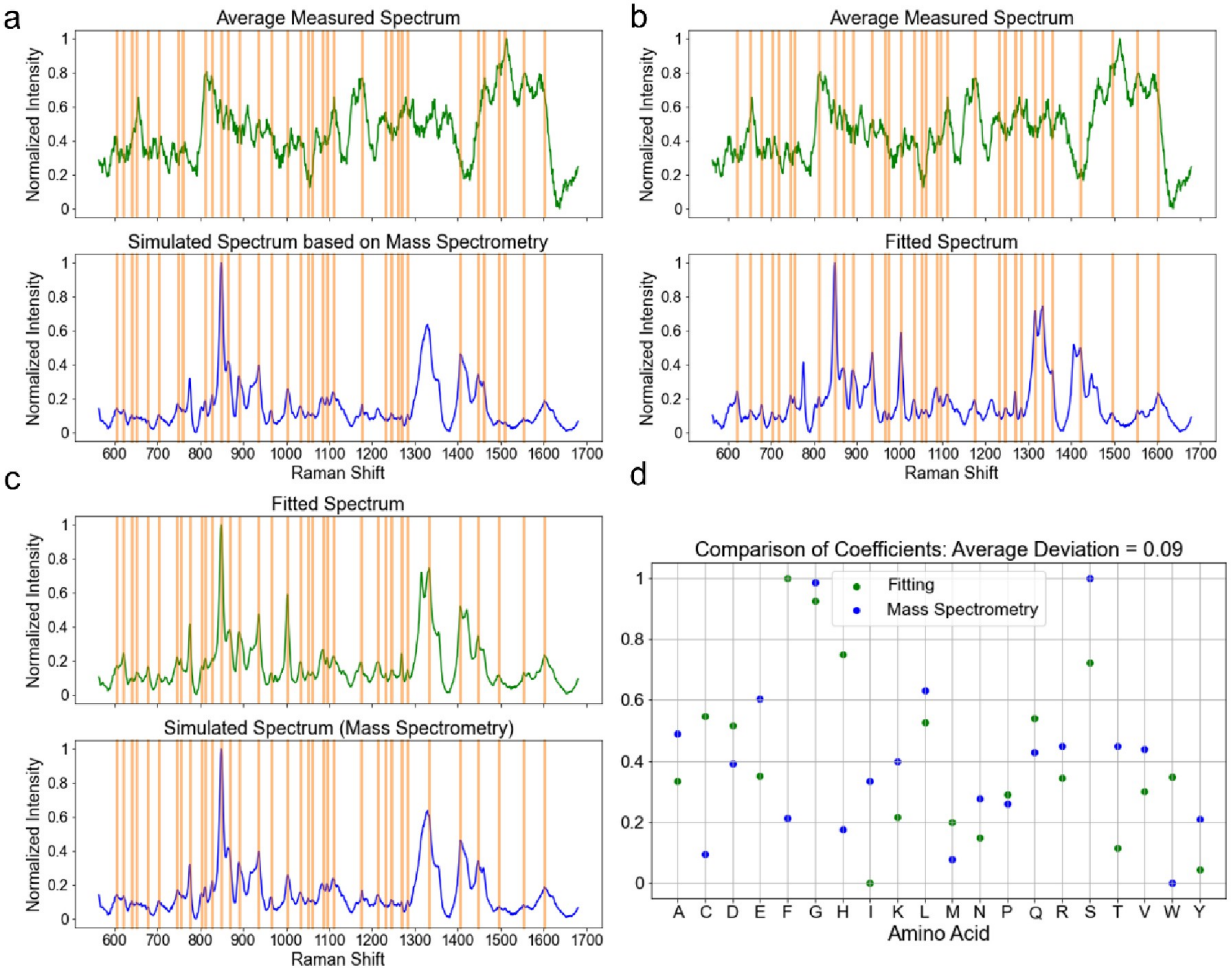
Comparison between the SERS data and MS data. (a) peak location comparison between the averaged spectrum from the spectra measured from 68 vesicles (HEK293+HRAS F8) and the simulated spectrum; (b) peak location comparison between the fitted spectrum and averaged measured spectrum; (c) peak location comparison between the fitted spectrum and the simulated spectrum; (d) comparison of the coefficients assigned for the 20 amino acids between the fitted and simulated spectra.

Each presented spectra file consists of an average of 75 spectra collected from the corresponding amino acid sample. Given the fact that different substances display uniquely different Raman interaction cross-sections, we next determined the relative SERS responses of all 20 amino acids by comparing the signal-to-noise ratios (SNR) of the SERS spectra, shown in the middle column of S1 Table in S1 File. In making sure to consider the relative natural amino acid abundance and relative SERS activities (S1 Table in S1 File), we present a simulated spectrum which mirrors the results of our proteomics analysis of the sEVs. In addition, we generated a “fitted” spectrum of an averaged spectrum measured from 51 individual sEVs derived from fraction 8 sEVs produced by HEK293+HRAS cells. The fitted spectrum was created from the spectra of the 20 pure amino acids pertinent to humans (a linear combination), by merely changing the multiply factor for each of the 20 spectra to fit the measured spectrum qualitatively. No information from MS about the relative abundance of proteins was included when generating the fitted spectrum. Fig 3a displays a comparison between the simulated spectrum derived from MS data and the averaged SERS spectrum derived from SERS spectra of individual sEVs. The qualitative matching rate for the data was 89% from the perspective of their peak locations. As shown in Fig 3b, the reported qualitative matching rate corresponded to 89% of the fitted spectrum and the averaged SERS spectrum of individual sEVs. Similarly, the matching rate increased to 94% when comparing the simulated and fitted spectrum, as shown in Fig 3c. By comparing the “assigned” relative amino acid abundances derived from the fitted spectrum to the experimentally MS-derived abundances of amino acids, the averaged deviation corresponded to 9% (Fig 3d). When comparing results derived from SERS and proteomics analyses, we observed a high consistency, demonstrating that SERS spectra collected from individual sEVs faithfully reflected the biomolecular composition of the sEVs. As mentioned above, the fitting process carried out here, as a qualitative study, was focusing on the peak locations rather than absolute peak intensities due primarily to the unavailability of the quantitative information of the proteins measured by MS.

We noted that laser diffraction and background caused by PBS crystals could bring down signal-to-noise ratio. With the future effort to improve the measurement condition and throughput/EV concentration, data analysis could involve just the spectra with high signal-to-noise ratio and still hold statistical significance. Nonetheless, in our current study, the data suggested that the spectral features could be extracted from the SERS spectra of the vesicles for comparison. We also acknowledge the existence of mismatches in our comparative data, especially those derived from peak intensities recorded from simulated and fitted-measured comparisons. In our view, such mismatches could legitimately be attributed to the presence of lipids and/or other elements (e.g., RNA & DNA) present in vesicles which are not expected to be located in the simulated spectrum only from amino acids. Nonetheless, the comparisons helped validate the utility of SERS spectra in determining the biomolecular compositions of sEVs with less than 10% deviation (on average) between the measured and the “assigned” amino acid abundance. Coupled with the MS data, the fitting results indicated that the spectral features detected were actually from the capsulated content of EV samples which paved the way for further spectral distinguishing. Our findings therefore help pave the way for future studies aimed at further resolving the link between sEV sizes and their biomolecular composition.

**Fig 4:**
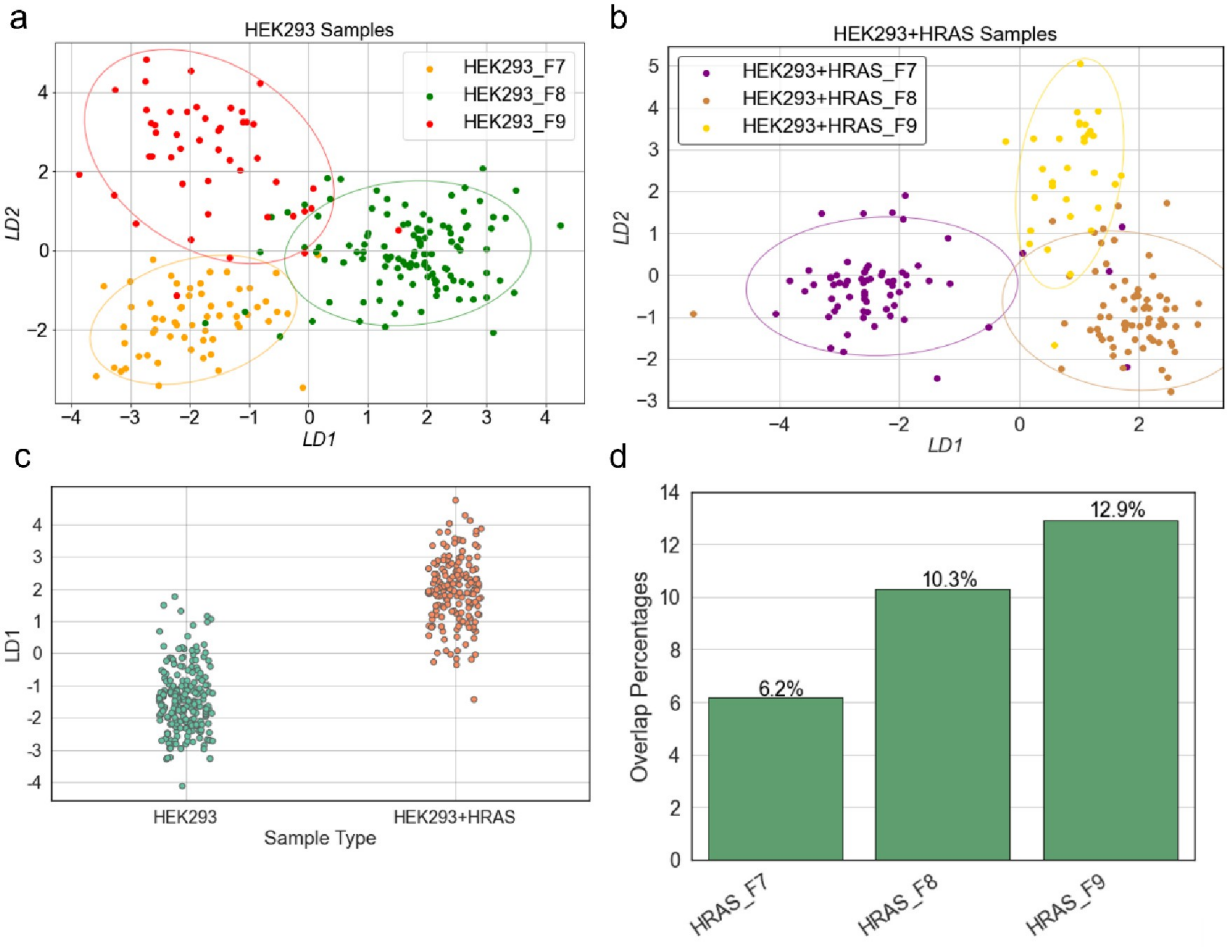
LDA analysis results of SERS signatures obtained from sEVs of different size groups. (a) LDA of the spectral signatures obtained from vesicles of HEK293 fractions 7, 8, 9 respectively; (b) LDA of the spectral signatures obtained from vesicles of HEK293+HRAS fractions 7, 8, 9 respectively; (c) Polling the SERS signature from the vesicles of the three size fractions from the HEK293 and HEK293+HRAS respectively; (d) The SERS signature overlapping rate of each fraction from the HRAS group.

Data shown in Fig 4 presents the biomolecular composition from HEK293- and HEK293+HRAS-derived vesicles as determined from their SERS spectra. The data was collected using sEVs that had been isolated into three different fractions (fractions 7–9) based on their elution prolife from the SEC column shown in Fig 2b & 2c. The number of spectra collected by SERS from HEK293-derived fractions 7, 8, and 9 consisted of 59, 115, and 47 respectively. Similarly, the number of HEK293+HRAS derived vesicles interrogated by SERS consisted of 65, 68, 31 data spots respectively. The assessment of biochemical contents in vesicles was carried out by comparing the spectral overlaps among the SERS spectra collected from individual sEVs. Results were projected onto a two-dimensional map using our LDA algorithm, as shown in Fig 4. Each point displayed in the plot represents a SERS spectrum recorded from an individual vesicle. The spectral variations between the two types of vesicles were measured by their Euclidean distances. In this case, the spectral difference was revealed by the magnitude of the distance between the data points. As shown in Fig 4a, the LDA map comparing the SERS spectra collected from HEK293-derived sEVs exhibited some level of internal spectral difference within each of the fractions. These data reveal the magnitude of differences in the biochemical content among individual vesicles. Importantly, when comparing the LDA maps between vesicles of the three fractions, no significant overlap was detected, suggesting that the external spectral differences among the three groups were larger than the spectral differences among individual sEVs within each group. The data demonstrate that size-based SEC fractionations of a bulk preparation of HEK293 cell-derived EVs resulted in the purification of homogeneous classes of sEVs with a defined and non-overlapping biomolecular composition as determined by SERS analysis. Furthermore, a similar pattern emerged when analyzing the SERS spectral features of HEK293+HRAS cell-derived vesicles using our LDA algorithm, shown in Fig 4b. In general, based on the LDA maps, less than 16% of the vesicle spectra were found “common” between different size-based fractions. It should be noted that the Raman laser spot size is larger than the vesicle diameters and that vesicles are invisible due to the instrumental limitation. Although our SEM images did not suggest that vesicles cluster within the nearest-spacing of 1 μm, there is a non-zero probability that occasional ones could be derived from more than one vesicle. Nonetheless, the data indicated that our measurements were based on single vesicles. In addition, despite our best practice, current techniques for sEV isolation and the protocol of SERS scanning resulted in less-than-optimal throughput. Such studies would undoubtedly benefit from improved vesicle concentration and SERS measurement throughput from statistical perspectives.

Interestingly, our data demonstrate that sEV groups within distinct fractions can be differentiated based on their biomolecular composition through their SERS spectral signature. The data suggesting the existence of “common” vesicles that are present across fractions likely derives from limitations associated with SEC to fully resolve sEVs exclusively according to distinct sizes. Indeed, our data demonstrate the existence of such vesicles, which are similarly sized but elute across different fractions. These observations highlight limitations associated with the use of current SEC methodologies for the isolation of distinct sEV subpopulations. Given the situation, our single-vesicle SERS platform offers an opportunity to further characterize the variation in biomolecular composition among individual sEVs within a subpopulation of vesicles isolated within a SEC sub-fraction. Our methodology thus addresses a gap in the field of sEV biology that is limited to bulk and/or specific marker-based analysis.

Lastly, to evaluate the distribution of individual sEVs within our comparison of HEK293- and HEK293+HRAS-derived vesicles, we generated an LDA map by pooling the SERS spectra contributed by all three fractions of both cell types to generate data shown in Fig 4c. This approach revealed an overlap between the two clusters, suggesting that the HRAS transgene expressed in HEK293+HRAS cells caused the releases of vesicles with both similar and different biomolecular composition compared to those released by HEK293 cells. By retrieving the spectral sources of the dots produced from the HEK293+HRAS sEV group that had LD scores within the range of those produced from the HEK293 sEV group, we generated a comparison noting the distribution of the individual vesicles (Fig 4d). Using this approach, we noted that the overlapping rates increased from 6.2% to 12.9% across fractions F7 to F9 in the HRAS group. This finding suggests that the biological perturbations caused HRAS expression in HEK293 cells results in altering the biomolecular composition of a defined population of sEVs released by the cells. However, a substantial portion of the sEVs shared similar biomolecular compositions with the ones released by HEK293 control cells.

Compared to other studies that have analyzed EVs using SERS, including but not limited to the ones mentioned previously^56-61^, our study focused on the subpopulations of the sEVs isolated based on SEC. We also provide experimental data indicating that the origins of the SERS spectral features are from the biomolecules of the vesicles by linking the results between SERS and MS. Collectively, results of this study support the value of our single-vesicle based SERS platform to explore the biomolecular composition of individual sEVs of different sizes and/or are released by different parental cells. Currently, such information is yet to be established and recognized by the community. The fact that our platform is capable of distinguishing vesicles released by two types of cells with only one gene alternation underscores the potential of this system for disease screening, diagnosis and monitoring. A limitation of this single vesicle analysis system includes the low throughput capacity for sEV scanning by SERS. Current work in our laboratory aims to improve both the areal density available to the vesicles when introduced to the substrate and the efficiency of the SERS measurement process. Nonetheless, our results illustrate the value of our SERS platform as a sensitive sEV detecting technique that can differentiate nano-sized vesicles based on their biomolecular composition at the single vesicle level. Our approach overcomes current challenges frequently associated with population averaging in the study of bulk EV biology. Our technology could therefore not only benefit sEV-based disease diagnosis/screening, but also facilitate further studies investigating the biogenesis and activity of sub-fractionation of vesicles released by the same and/or different parental cells.

## Conclusions

Findings from our study validate the use of SERS for investigating the biomolecular composition of small extracellular vesicles (sEVs) produced by cultured cells. In using this approach, we uncovered a strong correlation between the sEV size and their biomolecular composition. Specifically, 16% or less vesicles in each of the individual fractions displayed an overlapping biomolecular composition with sEVs of different fractions. The < 10% deviation of data derived from sEV analysis by SERS and mass spectrometry supports the robustness of SERS as a method to fingerprint the biomolecular composition of sEVs. Our work indicates that size-based fractions of the particular sEVs examined are indeed correlated with their respective biochemical contents as reflected by their SERS spectra. Future studies using SERS will improve our ability to investigate the biogenesis, diversity and functional consequences of vesicles released by cultured cells and those in more complex biofluids.

## Supporting information

S1 File Supporting figures and table. (DOCX)

S2 File Western blot data #1. NG IZON F6-9 DGUC F6-9 CD81_raw image.
(TIF)

S3 File Western blot data #2. HRAS IZON and DGUC F4-11 CD81_raw image.
(TIF)

S4 File Raw Raman data. Supporting_Raman data.
(RAR)

S5 File Mass spectrometry data. HEK293+HRAS_IZON_SEC_fraction_8_MS.
(XLSX)
