# Supplementary material for: Label-free single-vesicle based surface enhanced Raman spectroscopy: A robust approach for investigating the biomolecular composition of small extracellular vesicles
Source: PLoS One. 2024 Jun 18;19(6):e0305418. doi: 10.1371/journal.pone.0305418 (PMC11185487; doi:10.1371/journal.pone.0305418)
Supplement: S1 File — (DOCX) [file pone.0305418.s001.docx]

**Supporting Information:**

**Label-Free Single-Vesicle Based Surface Enhanced Raman Spectroscopy: A Robust Approach for Investigating the Biomolecular Composition of Small Extracellular Vesicles**

*Zirui Liu^1+^, Martin Ng^2+^,* *Siddharth Srivastava^1^, Tieyi Li^1^, Jun Liu^1^, Tuan Anh Phu^2^, Bogdan Mateescu^3,4^, Yi-Ting Wang^5^, Chia-Feng Tsai^5^, Tao Liu^5^, Robert L. Raffai^6,7#^, and Ya-Hong Xie^1,8#*^*

^1^Department of Materials Science and Engineering, University of California Los Angeles, Los Angeles, CA 90095, USA

^2^Northern California Institute for Research and Education, San Francisco, CA 94121, USA

^3^Brain Research Institute, University of Zürich, Winterthurerstrasse 190, CH-8057, Zürich, Switzerland

^4^Institute for Chemical and Bioengineering, ETH Zürich, Vladimir Prelog Weg 1, Zürich, 8093, Switzerland.

^5^ Biological Sciences Division, Pacific Northwest National Laboratory, Richland, WA 99354, USA

^6^Department of Surgery, Division of Vascular and Endovascular Surgery, University of California San Francisco, San Francisco, CA, 94143, USA.

^7^Department of Veterans Affairs, Surgical Service (112G), San Francisco VA Medical Center, San Francisco, CA 94121, USA

^8^UCLA Jonsson Comprehensive Cancer Center, University of California, Los Angeles, Los Angeles, CA 90095, USA

^+^ equal contribution

^#^ equal senior contribution

*To whom correspondence should be addressed:

Ya-Hong Xie

E-mail address: [yhx@ucla.edu](mailto:yhx@ucla.edu)

**Table of Contents**

1. Gold-nanopyramid SERS substrate fabrication
2. sEV isolation and NTA analysis
3. Immunoblotting
4. MS-based proteomics analysis
5. Table S1. Coefficients assignments of the 20 amino acids for generating the simulated spectrum
6. Figure S1. NTA result of HEK293-derived sEVs IZON SEC fraction 7
7. Figure S2. NTA result of HEK293-derived sEVs IZON SEC fraction 8
8. Figure S3. NTA result of HEK293-derived sEVs IZON SEC fraction 9
9. Figure S4. NTA result of HEK293+HRAS-derived sEVs IZON SEC fraction 7
10. Figure S5. NTA result of HEK293+HRAS-derived sEVs IZON SEC fraction 8
11. Figure S6. NTA result of HEK293+HRAS-derived sEVs IZON SEC fraction 9
12. Figure S7. SERS spectra of 20 amino acid, each averaged from 75 individual spectra

**Gold-nanopyramid SERS substrate fabrication**

A single layer of self-assembled polystyrene (PS) balls (⌀ 500 nm) was generated on a surface of DI water using the Langmuir–Blodgett patterning. The layer was then transferred to a 4” (001) silicon wafer with a layer of 50 nm SiO_2_ deposited on top using e-beam deposition. The PS balls were later removed using chloroform, at room temperature, after a deposition of 50nm Cr by e-beam. The exposed SiO_2_ were etched using reactive ion etching (25 sccm Ar, 25 sccm CHF3, RIE power 200W, etch time 2min) to selectively expose Si. Next, the exposed silicon was etched using KOH (60 wt% for 2 min at 60 °C). Inverted nanopyramids with sidewalls at 57.5-degree angles were created because of different etching rates along the [001] and [111] directions of silicon. The mold was finished by removing the residual Cr and SiO_2_ using 48% HF solution. Then, 200 nm of gold film was deposited by e-beam onto the pitted surface by electron beam deposition and bonded to a carrier wafer using epoxy before lifting off.

**sEV isolation and NTA analysis**

HEK293 cells carrying a doxycycline-inducible transgene, expressing mNeonGreen or HRAS-mNeonGreen fusion protein, were cultured in Pro293a Chemically Defined Medium (Lonza) supplemented with 1% Penicillin/Streptomycin (Gibco), 1% GlutaMAX (Gibco), and 1% fetal bovine serum (Gibco). Cells were cultured in 225 cm^2^ cell culture flask (Corning) until they reached 90% confluency. The cells were then washed twice with phosphate buffered saline (PBS) (Corning) and cultured in serum-free Pro293a Chemically Defined Medium (Lonza) supplemented with 1% Penicillin/Streptomycin (Gibco), 1% GlutaMAX (Gibco), and 1 ug/mL of Doxycycline (Sigma Aldrich). The conditioned media was collected after 48 hours.

For sEV isolation, the conditioned media was first centrifuged at 400 x g for 10 min at 4°C to pellet debris and dead cells, then centrifuged at 2000 x g for 20 min at 4°C to pellet large vesicles and leftover debris. The supernatant was filtered (0.2 μm) and centrifuged on 2 mL of a 60% iodixanol cushion (Stem Cell Technologies) at 100,000 x g for 3 hours (Type 50.2 Ti, Beckman Coulter). The resulting concentrated cushion (2 mL) was extracted with an extra 1 mL and further purified by loading onto a 35 nm qEV column (IZON), with PBS as the diluting buffer. Afterwards, 13 distinct fractions were collected. Particles in fractions 7, 8, and 9 were subjected to size and concentration measurement by Nanosight LM14 (Malvern Instruments, Westborough, MA) performed using a 488 nm detection wavelength. The analysis settings were optimized and standardized for each sample. Samples were diluted in either 1:100 or 1:200 PBS and measured in triplicates. The detection threshold was set at 3, and 3 videos (1 min long each) were captured to give the mean, mode, median, and estimated concentration for each sample. Data were analyzed using the NTA 3.3 software.

**Immunoblotting**

Each fraction of the IZON SEC purified sEVs (37.5 μL) was mixed with 12.5 μL of 4x Laemmli buffer (Bio-Rad) and heated at 95°C for 5 minutes. Samples were then loaded on a 4-20% SDS-PAGE gel and transferred onto a PVDF membrane (Bio-Rad). The membranes were blocked with 5% non-fat milk dissolved in PBS for one hour, and then were probed with anti-CD81 (1:500, Santa Cruz Biotechnology). After 4 washes in PBS containing 0.1% Tween (PBST), the membranes were incubated with HRP-conjugated secondary antibodies: anti-Mouse IgG-HRP (1:1000, Santa Cruz Biotechnology) for 1 hr and washed with PBST. Signals were visualized after incubation with Amersham ECL Prime substrate and imaged using an ImageQuant LAS 4000.

**MS-based proteomics analysis**

HEK293+HRAS sEV samples were first lysed using 5% SDS. Protein solutions were denatured with 10 mM DTT for 15 mins at 37 °C and alkylated with 50 mM iodoacetamide in the dark for 15 mins at room temperature. Afterward, the sample was added a final concentration of 2.5% phosphoric acid and then six volumes of binding buffer (90% methanol; 100 mM triethylammonium bicarbonate, TEAB; pH 7.1). After mixing, the protein solution was loaded to an S-Trap filter (ProtiFi), spun at 10000g for 1 min and then the filter was washed with 150 μL of binding buffer for 3 times. Finally, 1 μg of Lys-C and sequencing-grade trypsin and 20 μL of digestion buffer (50 mM TEAB) were added into the filter and the sample was digested at 37 °C for 16 h. To elute the peptides, 40 μL of 50 mM TEAB, 40 μL of 0.2% formic acid in H_2_O, and 40 μL of 80% acetonitrile in H_2_O were added sequentially. The peptide solutions were pooled and quantified BCA protein assay (Thermo Fisher Scientific). The peptides were dried with SpeedVac and stored at −80 °C until LC-MS/MS analysis.

The sEV peptides were reconstituted in 12 μL of 0.1% TFA with 2% ACN containing 0.01% DDM to reach a final concentration of 0.1 μg/μL, and 5 μL of the resulting sample was analyzed by LC-MS/MS using an Orbitrap Fusion Lumos Mass Spectrometer (Thermo Scientific) connected to a nanoACQUITY UPLC system (Waters) (buffer A: 0.1% FA with 3% ACN and buffer B: 0.1% FA in 90% ACN). Peptides were separated on an analytical column (75 μm i.d. × 20 cm) packed using 1.9-μm ReproSil C18 and with a column heater set at 48 °C, using an LC gradient (buffer A: 0.1% FA with 3% ACN and buffer B: 0.1% FA in 90% ACN): 2-6% buffer B in 1 min, 6-30% buffer B in 84 min, 30-60% buffer B in 9 min, 60-90% buffer B in 1 min, and finally 90% buffer B for 5 min at 200 nL/min. Data were acquired in a data-dependent acquisition mode and the peptides were isolated using a quadrupole system (the isolation window was 0.7). Ionized peptides with a mass range of 350-1650 m/z were scanned at 60,000 resolutions with maximum injection time (IT) of 50 ms and 100% automatic gain control (AGC) target (4E5). Precursor ions with intensities > 1E4 were selected for fragmentation by higher-energy collisional dissociation (HCD) at 30% collision energy and scanned in an orbitrap with a 100% AGC (5E4) and an IT of 300 ms.

The raw MS/MS data were processed with MSFragger via FragPipe.^1, 2^ The MS/MS spectra were searched against a human UniProt database (fasta file dated July 31, 2021, with 34,386 sequences which contain 17,193 decoys) and (initial) fragment mass tolerances were set to 20 ppm. A peptide search was performed with full tryptic digestion (Trypsin) and allowed a maximum of two missed cleavages. Carbamidomethyl (C) was set as a fixed modification; acetylation (protein N-term), and oxidation (M) were set as variable modifications. For match-between-run (MBR) analysis, 10 ppm m/z tolerance, 1.5 mins RT tolerance and 0.05 MBR ions FDR were used for analysis. The final reports were then generated (peptide-spectrum match (PSM), ion, peptide, and protein) and filtered at each level (1% protein FDR plus 1% PSM/ion/peptide-level FDR). The intensities of each protein/peptide were extracted from FragPipe outputs.

**Table S1. Coefficients assignments of the 20 amino acids for generating the simulated spectrum**


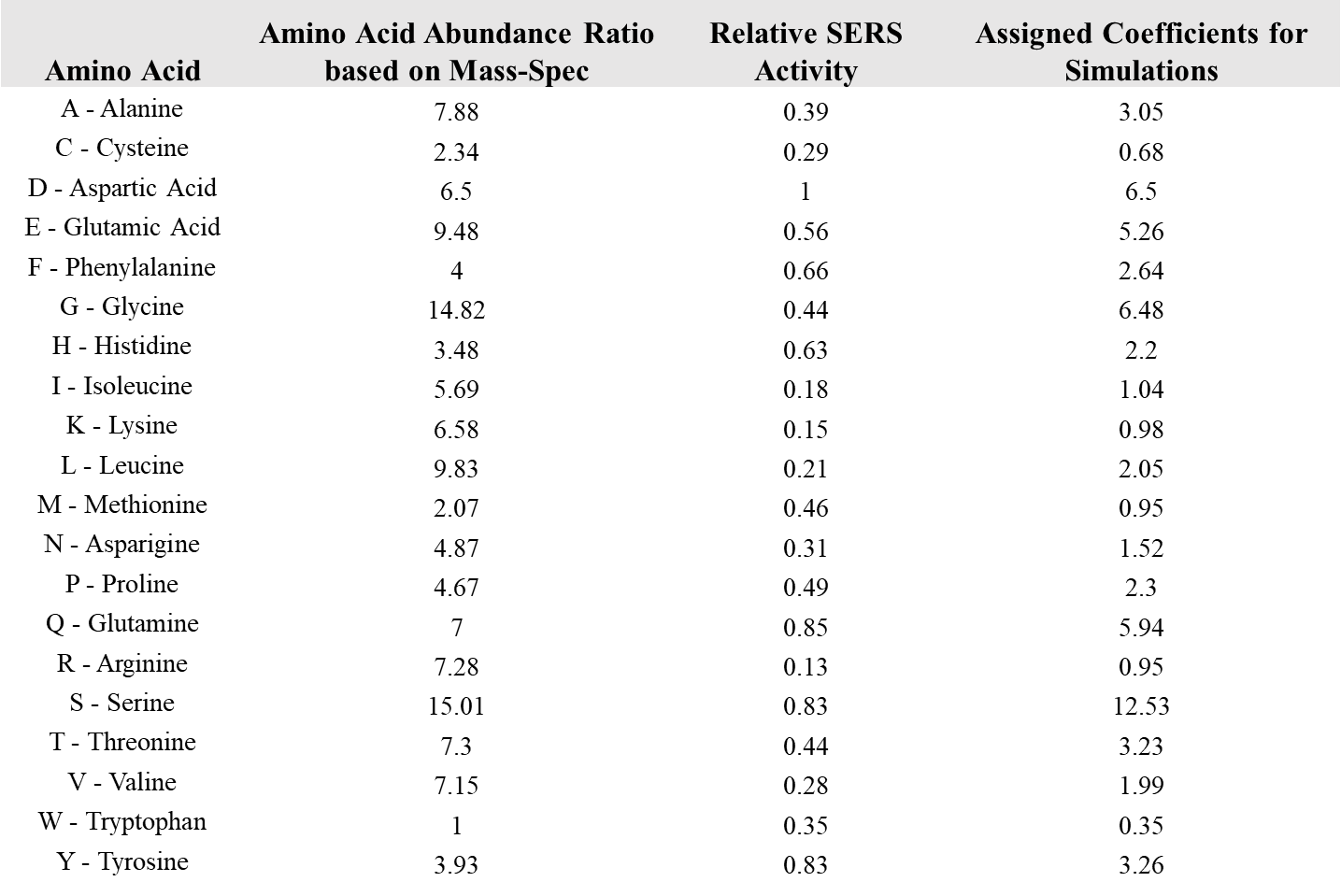


**Figure S1. NTA result of HEK293-derived sEVs IZON SEC fraction 7**


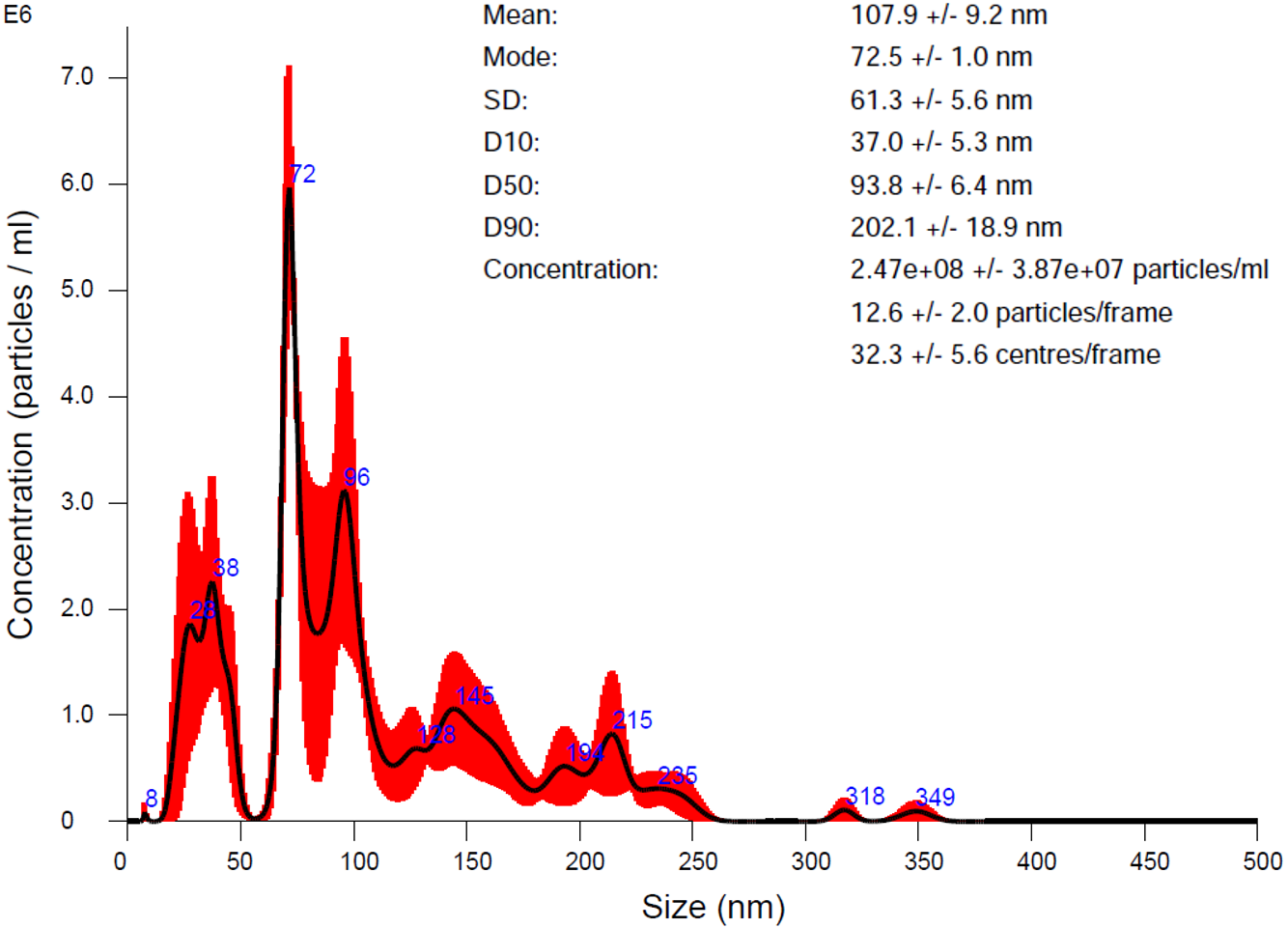


**Figure S2. NTA result of HEK293-derived sEVs IZON SEC fraction 8**
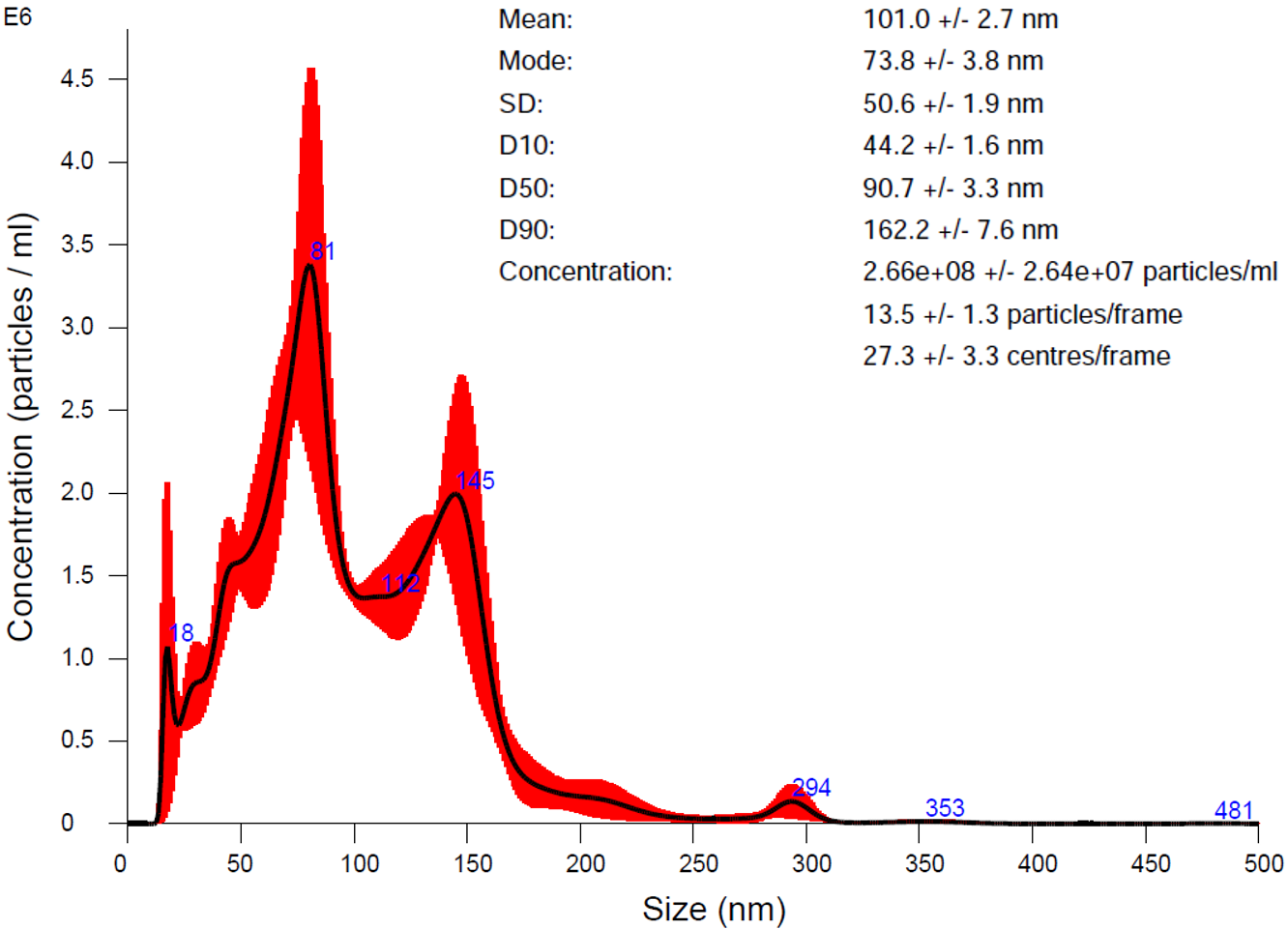


**Figure S3. NTA result of HEK293-derived sEVs IZON SEC fraction 9**
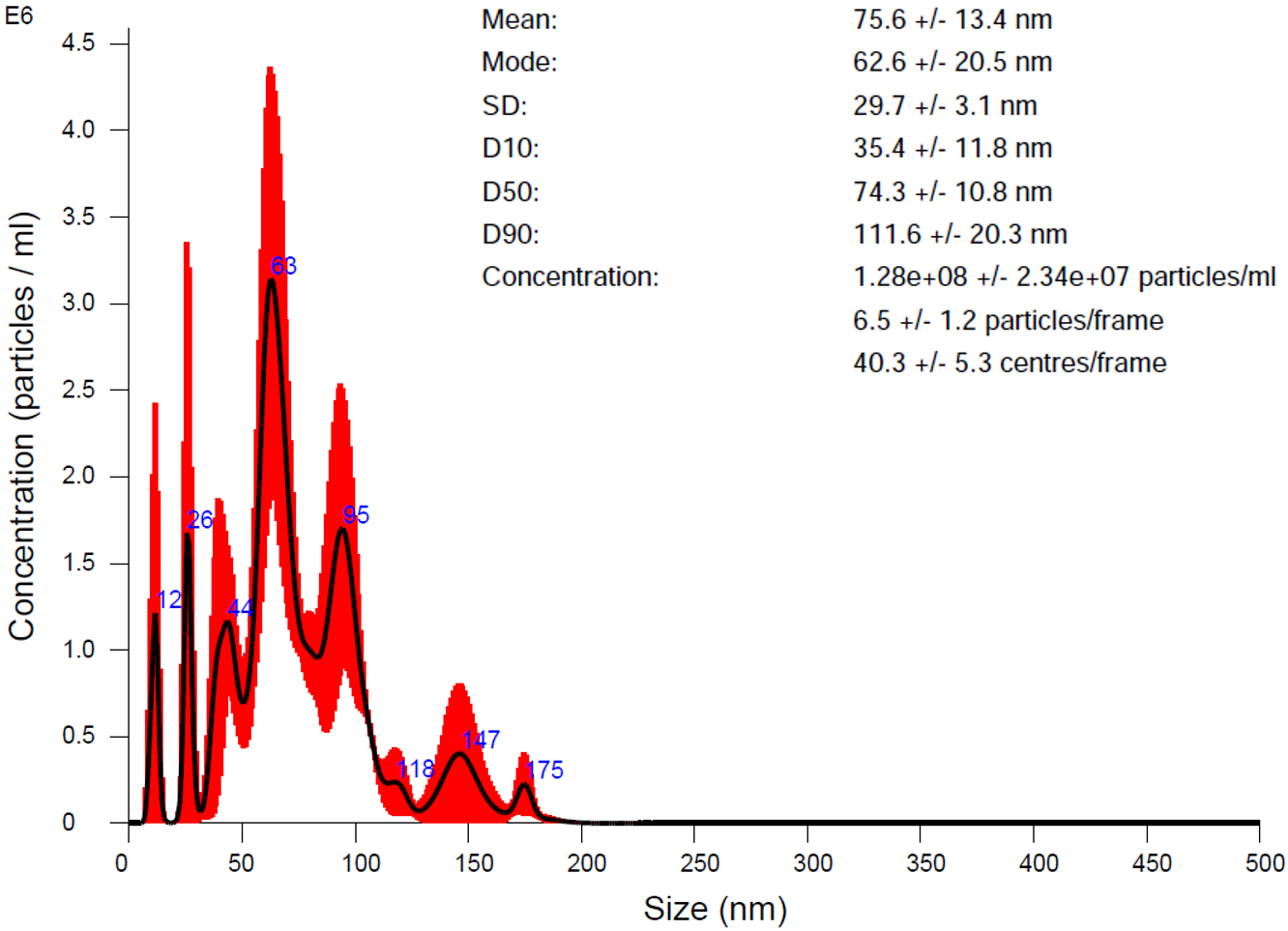


**Figure S4. NTA result of HEK293+HRAS-derived sEVs IZON SEC fraction 7**
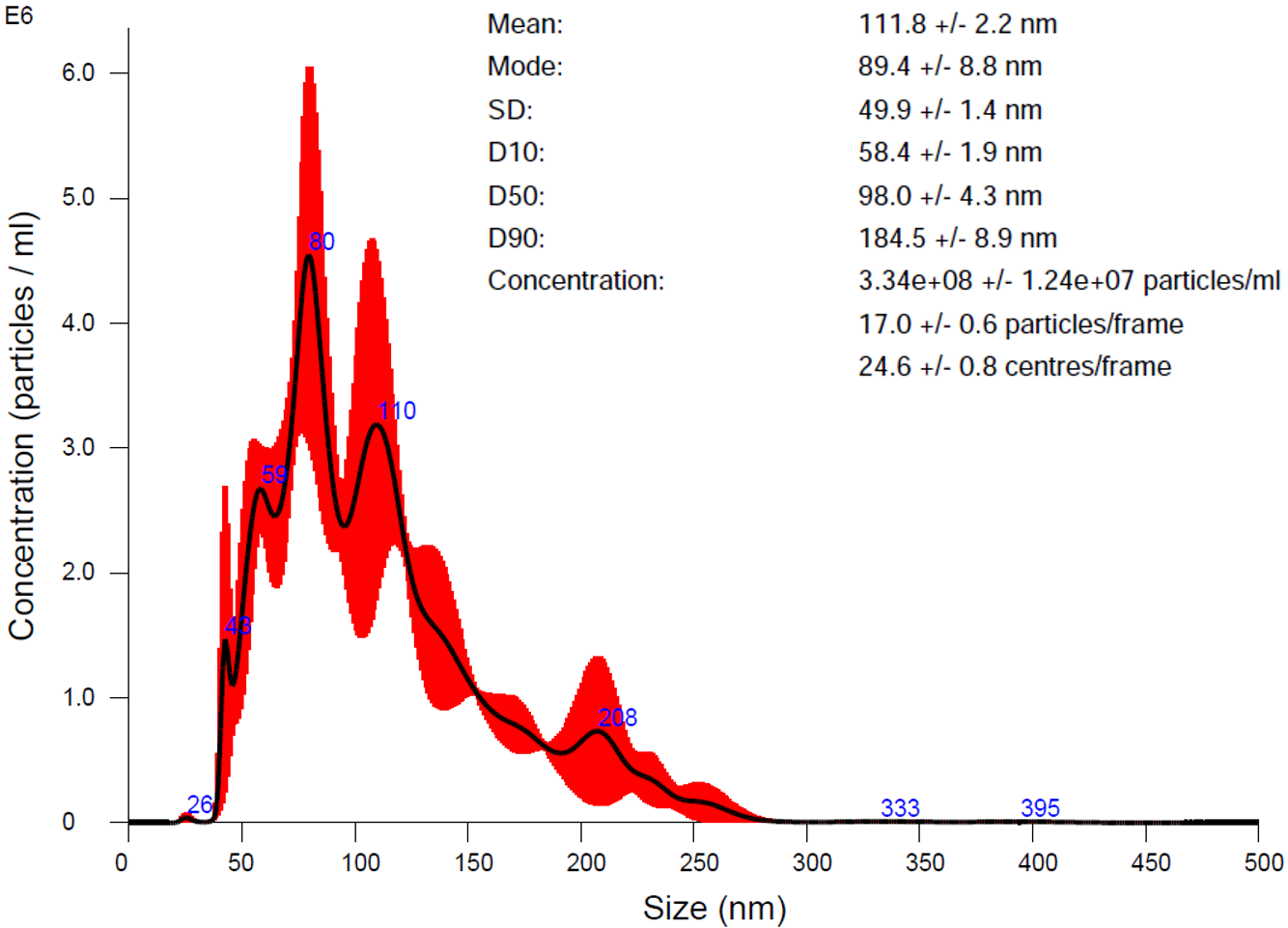


**Figure S5. NTA result of HEK293+HRAS-derived sEVs IZON SEC fraction 8**
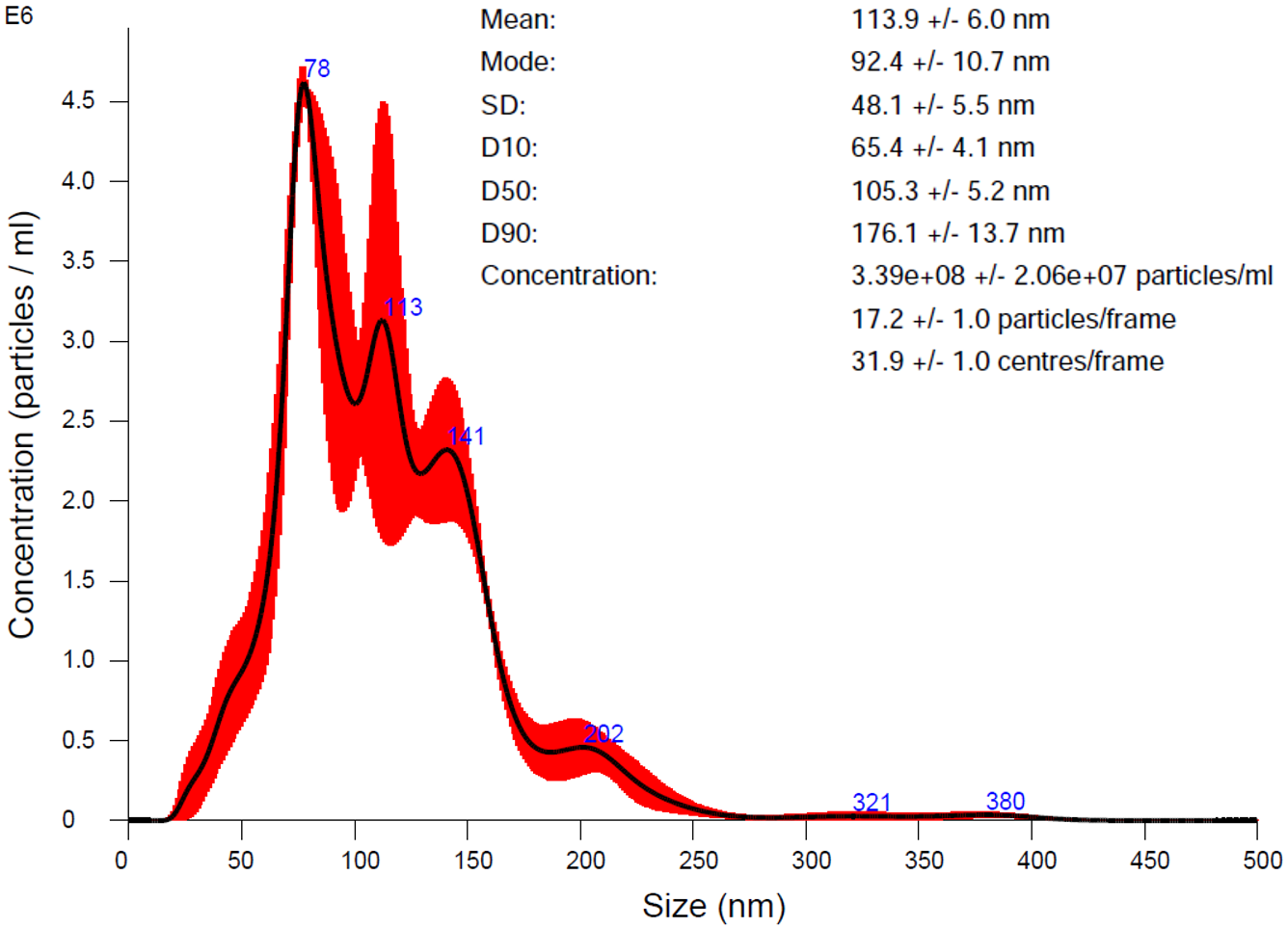


**Figure S6. NTA result of HEK293+HRAS-derived sEVs IZON SEC fraction 9**
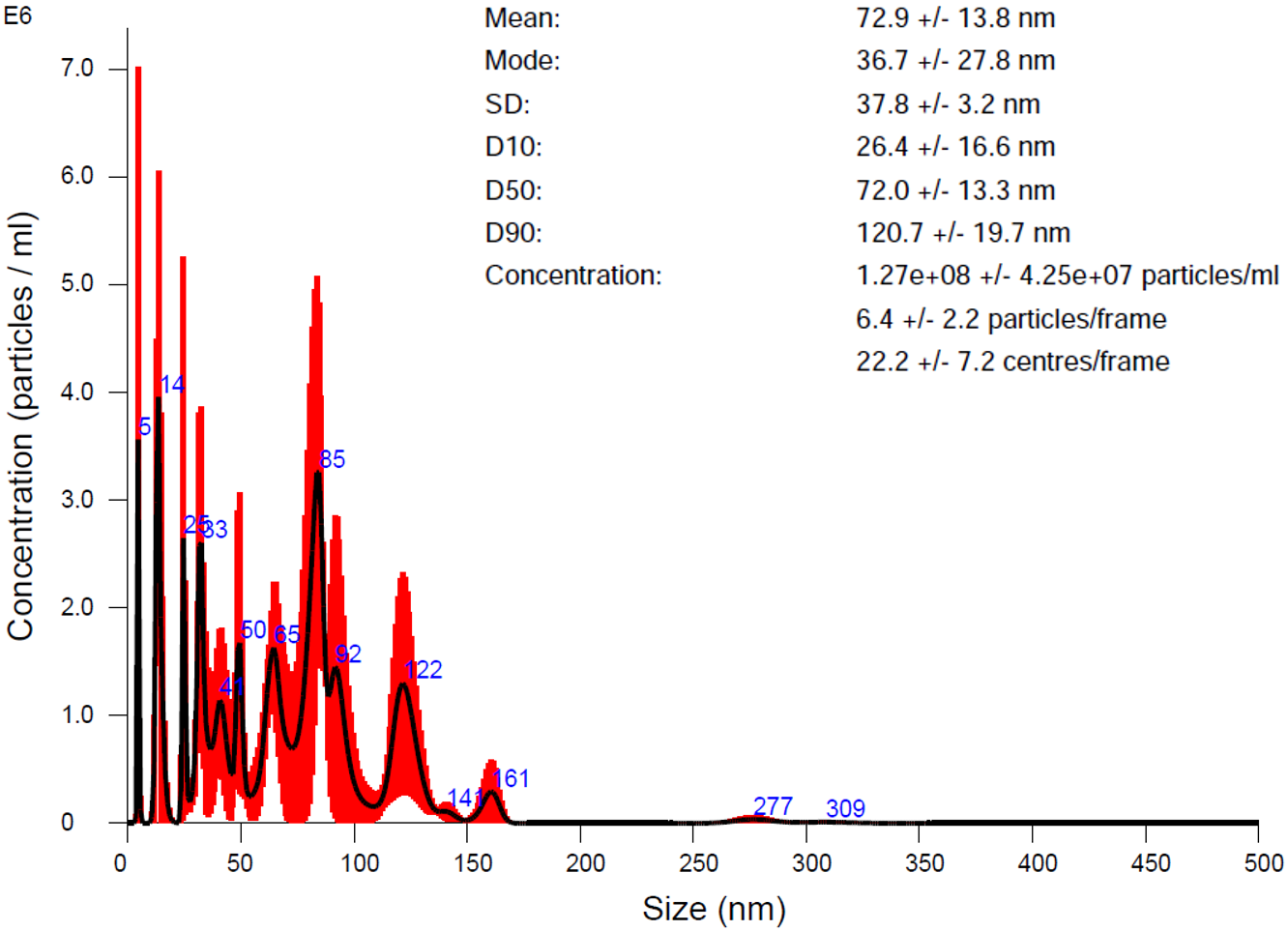


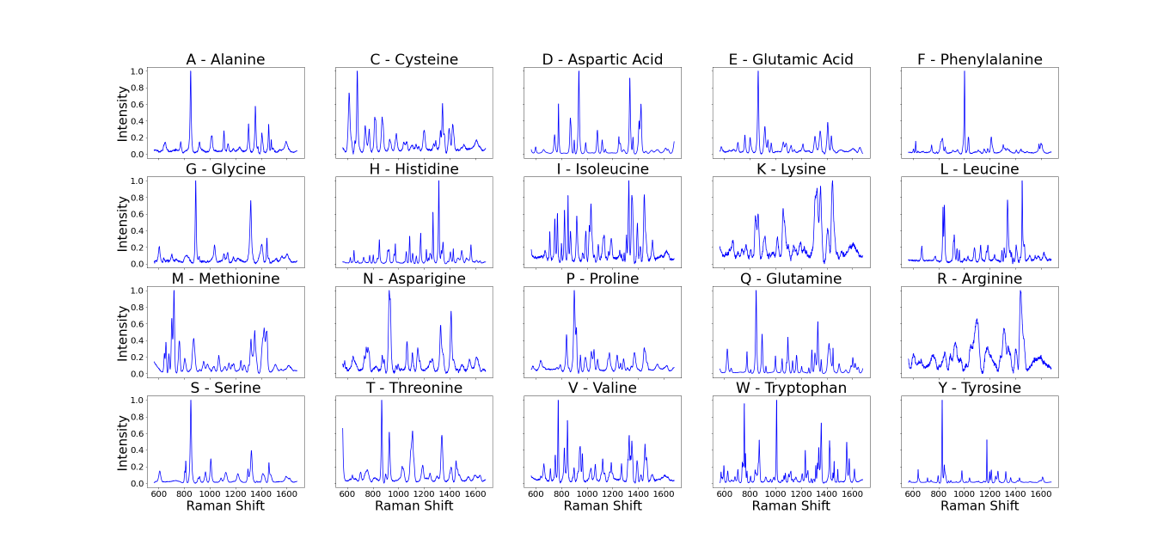
**Figure S7. SERS spectra of 20 amino acid, each averaged from 75 individual spectra**

**References**

1. Kong, A. T.; Leprevost, F. V.; Avtonomov, D. M.; Mellacheruvu, D.; Nesvizhskii, A. I., MSFragger: ultrafast and comprehensive peptide identification in mass spectrometry-based proteomics. *Nat Methods* **2017,** *14* (5), 513-520.

2. Teo, G. C.; Polasky, D. A.; Yu, F.; Nesvizhskii, A. I., Fast Deisotoping Algorithm and Its Implementation in the MSFragger Search Engine. *J Proteome Res* **2021,** *20* (1), 498-505.
