# Supplementary figures and images for: Label-free single-vesicle based surface enhanced Raman spectroscopy: A robust approach for investigating the biomolecular composition of small extracellular vesicles
Source: PLoS One. 2024 Jun 18;19(6):e0305418. doi: 10.1371/journal.pone.0305418 (PMC11185487; doi:10.1371/journal.pone.0305418)

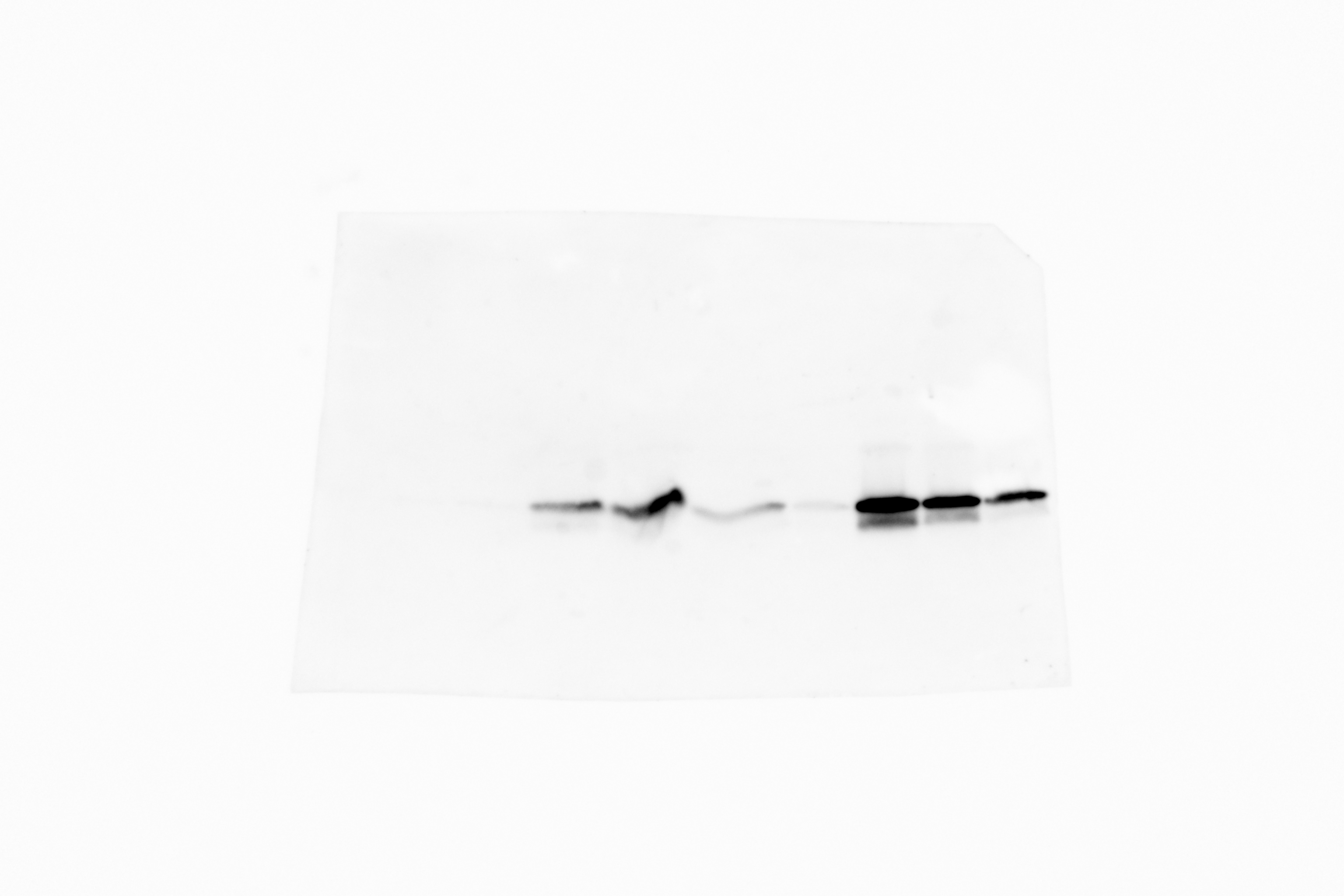

Supplement: S2 File — NG IZON F6-9 DGUC F6-9 CD81_raw image. (TIF) [file pone.0305418.s002.tif]

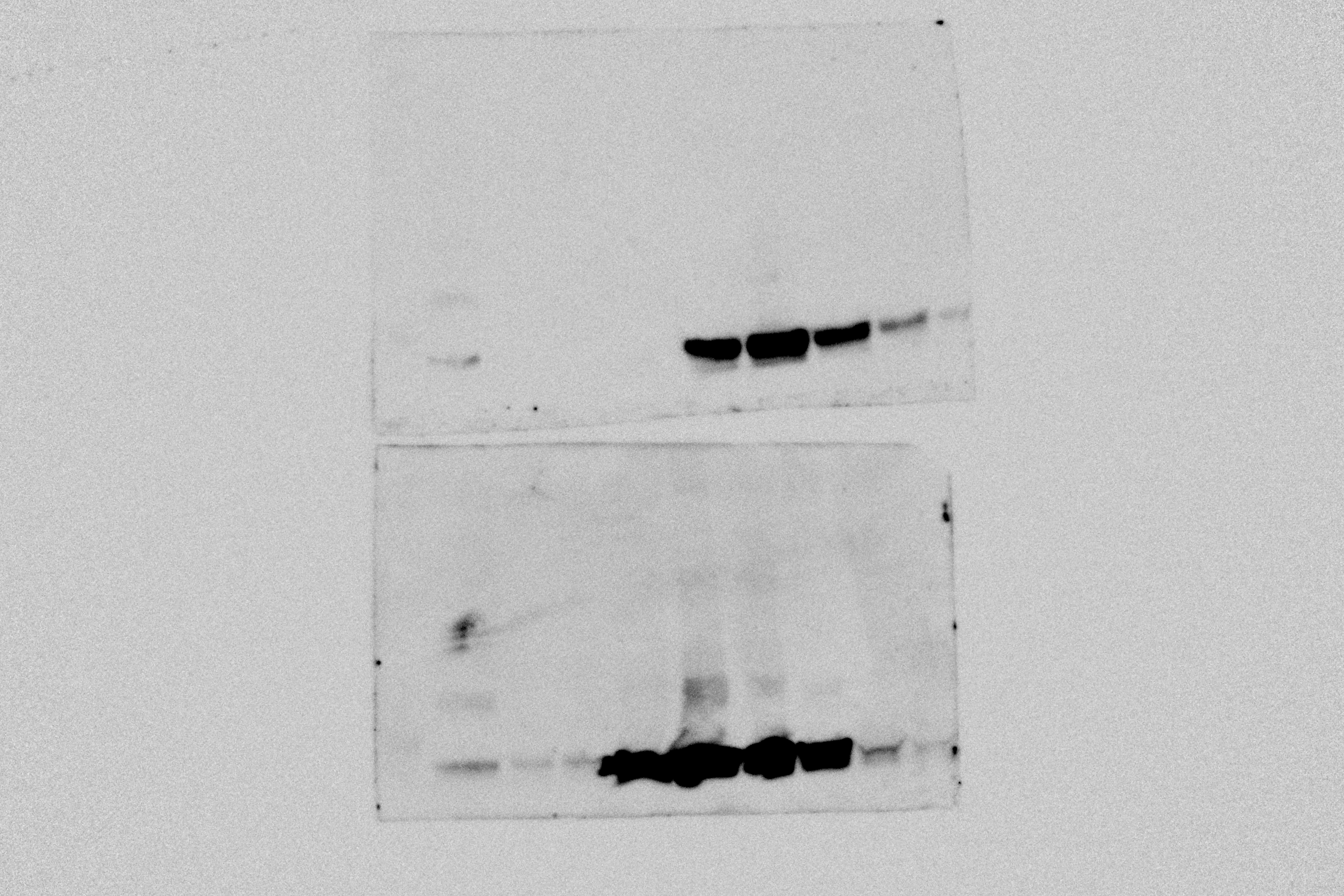

Supplement: S3 File — HRAS IZON and DGUC F4-11 CD81_raw image. (TIF) [file pone.0305418.s003.tif]
